# Down-regulation of SFRP1 as a putative tumor suppressor gene can contribute to human hepatocellular carcinoma

**DOI:** 10.1186/1471-2407-7-126

**Published:** 2007-07-12

**Authors:** Jian Huang, Yun-Li Zhang, Xiao-Mei Teng, Yun Lin, Da-Li Zheng, Peng-Yuan Yang, Ze-Guang Han

**Affiliations:** 1 Shanghai-Ministry Key Laboratory of Disease and Health Genomics, Chinese National Human Genome Center at Shanghai, and Department of chemistry of Fudan University, 351 Guo Shou-Jing Road, Shanghai 201203, China; 2 Rui-Jin Hospital affiliated to Jiaotong University, 197, Rui-Jin II Rd. Shanghai 200025, China; 3 Department of biochemistry, Taishan Medical College, 2 East Yingsheng Road, Taian, Shandong 271000, China

## Abstract

**Background:**

Hepatocellular carcinoma (HCC) is one of the most common cancers in the world. SFRP1 (the secreted frizzled-related protein 1), a putative tumor suppressor gene mapped onto chromosome 8p12-p11.1, the frequent loss of heterozygosity (LOH) region in human HCC, encodes a Wingless-type (Wnt) signaling antagonist and is frequently inactivated by promoter methylation in many human cancers. However, whether the down-regulation of SFRP1 can contribute to hepatocarcinogenesis still remains unclear.

**Methods:**

We investigated the expression of SFRP1 through real time RT-PCR and immunohistochemistry staining. The cell growth and colony formation were observed as the overexpression and knockdown of SFRP1. The DNA methylation status within SFRP1 promoter was analyzed through methylation-specific PCR or bisulphate-treated DNA sequencing assays. Loss of heterozygosity was here detected with microsatellite markers.

**Results:**

SFRP1 was significantly down-regulated in 76.1% (35/46) HCC specimens at mRNA level and in 30% (30/100) HCCs indicated by immunohistochemistry staining, as compared to adjacent non-cancerous livers. The overexpression of SFRP1 can significantly inhibit the cell growth and colony formation of YY-8103, SMMC7721, and Hep3B cells. The RNA interference against the constitutional SFRP1 in the offspring SMMC7721 cells, which were stably transfected by ectopic SFRP1, can markedly promote cell growth of these cells. LOH of both microsatellite markers D8S532 and D8SAC016868 flanking the gene locus was found in 13% (6 of 46 HCCs) and 6.5% (3 of 46 HCCs) of the informative cases, respectively, where 5 of 8 HCC specimens with LOH showed the down-regulation of SFRP1. DNA hypermethylation within SFRP1 promoter was identified in two of three HCC specimens without SFRP1 expression. Moreover, the DNA methylation of SFRP1 promoter was significantly reduced, along with the re-expression of the gene, in those HCC cell lines, Bel7404, QGY7701, and MHCC-H, as treated by DAC.

**Conclusion:**

Our data suggested that the down-regulation of SFRP1 as a candidate tumor suppressor gene, triggered by the epigenetic and/or genetic events, could contribute to the oncogenesis of HCC.

## Background

Hepatocellular carcinoma (HCC) is one of the most common cancers in the world, in particular in Sub-Sahara Africa and South-eastern Asia, with an estimation of 600,000 deaths annually [1,2]. The higher incidence rate of HCC has been continuing to increase in China and other countries over the past decades years [3-5]. The factors of susceptibility, such as the infection of hepatitis B virus (HBV) and hepatitis C virus (HCV), and chronic exposure to aflatoxin B1 (AFB1) and alcoholic cirrhosis, have been well identified and characterized as contributors to hepatocarcinogenesis. However, the underlying molecular mechanisms contributing to hepatocarcinogenesis are still unclear.

In past years, some transcriptomic approaches, such as expressed sequence tags (ESTs) and cDNA or oligonucleotide microarray, were employed to figure out the gene expression profile of HCC [6,7]. The comprehensive data will contribute to the understanding of oncogenesis and identification of some biomarkers related to the diagnosis and prognosis of HCC. Our previous transcriptomic data on HCC disclosed some deregulated genes in hepatocarcinogenesis as compared to that of the corresponding non-cancerous livers [8,9], of which some genes could contribute to the oncogenesis of HCC as HCC-associated genes, whatever activated oncogenes or silenced tumor suppressor genes. Among them, SFRP1 (secreted frizzled-related protein 1 gene), a putative tumor suppressor gene mapped onto chromosome 8p12-p11.1, a frequent loss of heterozygosity (LOH) region in human HCC [10], was found to be down-regulated in HCC. As known, some groups have reported that SFRP1 as a Wingless-type (Wnt) signaling antagonist is frequently inactivated owing to the promoter methylation in many human cancers [11-22]. Although SFRP1 can regulate the vascular cell proliferation, postnatal skeletal muscle growth and hypertrophy and the growth of retinal ganglion cell axons [23-26], whether the down-regulation of SFRP1 could contribute to the hepatocarcinogenesis should be further clarified.

In this study, we found that SFRP1 was significantly down-regulated in HCC specimens through epigenetic and/or genetic events, as compared to adjacent non-cancerous livers. Furthermore, the resulting data from RNA interference and cell transfection of exogenous SFRP1 indicated that the down-regulation of SFRP1 could contribute to hepatocarcinogenesis.

## Methods

### Tissue specimens

All HCC specimens were obtained from those patients who underwent surgical resection of their diseases and were informed consent before operation on their liver. The primary tumor specimens were immediately frozen at -80°C until DNA/RNA extraction. Both tumor and adjacent non-tumor tissues were sampled respectively, with approximate 1 cm^3 ^size of each specimen, and were proved by pathological examination. Those HCC specimens in this presenting work were grouped as the differentiation grades II-III according to the Edmondson grading system. The clinical characteristics of patients and tumors are summarized in Table 1. In addition, two adult liver tissues were obtained from two patients who were resected surgically due to hemangioma in liver. The samples were obtained from the portion unaffected by the hemangioma and frozen in liquid nitrogen immediately. The samples were sectioned and confirmed histologically. Two fetal livers were obtained from aborted fetus as the pregnancy between 4–6 months. All procedures and risks were explained verbally and in a written consent form. This project and protocol for the investigation involving human and animals were approved by the ethics committee of the Chinese National Human Genome Center at Shanghai.

**Table 1:** Summary of Analyses of the SFRP1 Gene in 46 HCC Tissues

Case ID	Folds	Flag	Gender	Age	HBV	HCV	Size(cm)	D8S532	D8SAC016868
1	0.66	-	M	40	+	-	3	-	-
2	1.36	-	M	45	+	+	2	+	-
3	0.18	+	M	50	+	-	3.5	-	-
4	0.67	-	M	37	+	-	11	-	-
5	0.44	+	M	55	+	-	4	-	-
6	0.29	+	M	60	+	-	3	-	-
7	0.12	+	M	65	+	-	7	-	-
8	0.05	+	M	50	+	+	16	-	-
9	7.09	-	M	50	+	-	16	-	-
10	0.06	+	M	50	+	-	16	-	+
11	0.01	+	M	40	+	-	5	+	+
12	0.04	+	M	34	+	-	11	-	-
13	0.28	+	F	50	+	-	4	-	-
14	0.11	+	M	52	+	-	9	+	-
15	0.52	-	M	53	+	-	4	-	-
16	0.01	+	M	46	+	-	5	+	-
17	0.02	+	M	48	+	-	12	-	-
18	0.01	+	M	41	+	-	3	-	-
19	0.00	+	M	60	+	-	3.5	+	-
20	0.23	+	M	45	+	-	15	-	-
21	5.15	-	M	41	+	-	10	-	-
22	0.14	+	M	49	+	-	5	-	-
23	0.23	+	M	30	+	-	3	-	-
24	0.15	+	M	44	+	-	4	-	-
25	1.26	-	M	65	+	-	11	-	-
26	0.17	+	M	37	+	-	18	-	-
27	0.31	+	M	49	+	-	9	-	-
28	0.04	+	M	75	+	-	2.5	-	-
29	0.06	+	M	44	+	-	3.5	-	+
30	0.19	+	M	53	+	-	5	-	-
31	0.43	+	M	41	+	-	4.5	-	-
32	0.36	+	M	61	+	-	14	-	-
33	1.37	-	M	70	+	-	3	-	-
34	0.57	-	F	48	+	-	5	-	-
35	0.41	+	M	48	+	-	4	-	-
36	2.54	-	F	59	+	-	6	-	-
37	0.01	+	M	51	+	-	5	+	-
38	0.03	+	M	51	+	-	4.5	-	-
39	1.52	-	F	33	+	-	15	-	-
40	0.17	+	M	53	+	-	6	-	-
41	0.08	+	M	50	+	-	5	-	-
42	0.31	+	M	42	+	-	6	-	-
43	0.04	+	M	58	+	-	12	-	-
44	0.06	+	F	51	+	-	4	-	-
45	0.19	+	F	55	+	-	8	-	-
46	0.44	+	M	58	+	-	12	-	-

Flag + indicated downregulation of SFRP1 gene (fold less than 0.5); – indicated no downregulation of SFRP

### Liver cancer cell lines

Liver tumor-derived cell lines (Bel7402, Bel7404, Bel7405, QGY7701, QGY7703, SMMC7721, Hep3B, HepG2, MHCC-L, MHCC-H, Sk-hep1, Huh-7, PLC, YY-8103 and Focus) and the fetal liver-derived cell line L02 were employed in this study, where MHCC-H and MHCC-L cells were kindly provided by the Cancer Institute affiliated to Zhongshan Hospital, Fudan University. All of these cell lines were grown under standard cell culture conditions in the following media: minimum essential medium Eagle (Sigma, Dorset, UK) supplemented with 10% fetal bovine serum (Life Technologies), 1% L-glutamine (L-glut) and 1% nonessential amino acids (NEAA) in a 5% CO_2_-humidified chamber.

### Extraction of genomic DNA and total RNA

In this work, genomic DNA was extracted from all available specimens using the DNeasy Tissue Kit (Qiagen, Valencia, CA) according to the manufacturer's recommendation. RNA was extracted using TRIzol solution according to the manufacturer's recommendation. And RNAse-free DNase I was used to remove DNA contamination. Total RNA concentration and quantity were assessed by absorbency at 260 nm using a DNA/Protein Analyzer (DU 530, Beckman, USA).

### Semi-quantitative RT-PCR and quantitative real-time RT-PCR

Reverse transcription (RT) was performed in a 20 μl reaction system with 2 μg total RNA treated by DNase I according to the manufacturer's recommendation. Each PCR was generally performed in 35 thermal cycles and then the PCR products were observed by electrophoresis on 2% agarose gel and visualized after staining with ethidium bromide, where β-actin was employed as loading control. The primers of SFRP1 were showed as following: forward, 5'-AAAGCAAGGGCCATTTA GATTAG-3'; reversal, 5'-TTCTGGGCTTGACCTTAATTGTA-3'. The PCR product is 328 bp. β-actin primers are: forward, 5'-TCACCC ACACTGTGCCCATCTACGA-3'; reversal, 5'-CAGCGGAACCGCTC ATTGCCAATGG-3'. The PCR product is 295 bp. To further analyze expression of SFRP1 gene in HCC specimens, the relative mRNA level of SFRP1 was measured by a quantitative real-time RT-PCR using TaKaRa PCR Thermal Cycler Dice Detection System and SYBR green dye (TaKaRa, Japan) in additional 40 paired HCC specimens according to the manufacturer's recommendation. A housekeeping gene, β-actin was used as an internal control. Measurements were repeated thrice to ensure the reproducibility of results. The mRNA level of each gene in each HCC sample was normalized by comparing with the level in corresponding non-cancerous liver [27]. The average Ct value of the β-actin gene was subtracted from the average Ct value of SFRP1 for each sample: SFRP1ΔCt = (Avg. SFRP1 Ct – Avg. β-actin Ct), SFRP1ΔΔCt= (SFRP1ΔCt_HCC -SFRP1ΔCt_non-HCC). The fold change (2^-SFRP1ΔΔCt^) of SFRP1 expression relative to β-actin of each HCC sample examined was calculated. The significance level was defined as p value <0.01 [28].

### Immunohistochemistry

Four-μm thick sections were deparaffinized and dehydrated, and then treated with methanol containing 0.3% H_2_O_2 _to inhibit endogenous peroxidase. The slides were incubated with rabbit anti-SFRP1 polyclonal antibody (1:100 dilution, 600-401-475, Rockland Inc.) at 37°C for 2 hrs, and then at 4°C overnight, followed by the incubation with a horseradish peroxidase-conjugated anti-rabbit antibody (Dako Japan Ltd., Kyoto, Japan) at 37°C for 1 h. The signals were detected using Diaminobenzidine Substrate Kit (Vector Laboratories, Burlingame, CA). Counterstaining was performed with hematoxylin. Slides incubated with PBS buffer instead of the primary rabbit antibody were used as negative controls, whereas normal liver in which SFRP1 was known to be strongly positive were used as positive controls in each experiment. In addition, the slides with HCC specimens and corresponding adjacent non-HCC livers were simultaneously examined via the immunohistochemistry staining, and then were assessed by visual inspection and the estimation of the percentage of immunopositive cells. The HCC specimens with less than 10% immunopositive cells were considered as negative. Tissues were graded on a scale of negative (-), low expression (+), high expression (++), or strong expression (+++). If ++ or +++ was observed, the specimen was considered to be strongly positive.

### Western blot analysis

Total proteins from cultured cell lines were subjected to protein gel electrophoresis using 12% SDS-PAGE and transferred onto Hybrid- PVDA membrane (Amersham life Science) treated by 20% methanol in Tris-glycine buffer. After blocked in PBS containing 5% BSA, the membrane was incubated for immunoblotting analysis with rabbit anti-SFRP1 polyclonal antibody (600-401-475, Rockland Inc.) by 1:300 dilutions at room temperature for 2 hrs, and then with goat-anti-rabbit secondary antibody for 40 min. Finally, the signals were detected using the Odyssey Infrared Imaging System (LI-COR Biosciences).

### Cell transfection and cell proliferation

All HCC cell lines were grown at 37°C in Dulbecco's modified Eagle's medium (Sigma Chemicals, St. Louis, Missouri, MO) supplemented with 10% fetal bovine serum (Life Technologies), in a 5% CO_2_-humidified chamber. To observe the cell proliferation, the recombinant plasmids pcDNA3.0 containing full-length ORF of SFRP1, which was amplified from a normal liver with high fidelity PCR Enzyme (PrimerStar, TaKaRa, Dalian, China), were transiently transfected into target cells using Lipofectamine™ 2000 Transfection Reagent (Invitrogen) according to the manufacturer's instruction. The transfected cells were seeded in 96-well plate at 2 × 10^3 ^cells per well and then cultured for 5–8 d. 10 μl of CCK-8 (Cell Counting Kit-8, Dojindo Laboratories, Kumamoto, Japan) solution was added to each well of per plate, and incubated the plate at 37°C for 1 h. The absorbance at 450 nm was measured to represent the cell viability. To establish the stable offspring cell lines with exogenous SFRP1, above plasmids and empty vectors as control were transfected into SMMC7721 cells and then G418 (Life Technologies, Inc., Paisley, UK) was added to the medium at a final concentration of 700 μg/ml. After 3 weeks, the remaining colonies were individually picked and expanded. The expression of exogenous SFRP1 in these offspring subclones was checked by western blotting using anti-SFRP1 polyclonal antibody. Cell viability was measured to assess the cell proliferation of those stable SMMC7721 subclones with exogenous SFRP1 according to the described method above. All experiments were independently repeated at least three times.

### Colony formation

Plasmids pcDNA3. 0 containing SFRP1 or empty vector as control were transfected into Hep3B and YY-8103 cells in 35-mm dishes by Lipofectamine 2000 (Invitrogen) for 24 hrs, and then stripped and plated on 100-mm tissue culture dishes, and then G418 (Life Technologies, Inc., Paisley, UK) was added to the medium at a final concentration of 700 μg/ml. After 3 weeks, the remaining colonies were counted on crystal-violet-stained dishes.

### Loss of heterozygosity (LOH) analysis

LOH analysis were here performed using DNA sequencing in 46 pairs of HCC and adjacent non-cancerous livers with the polymorphic microsatellite markers D8S532 (Forward primer: GCTCAAAGCCTCC AATGAC; Reverse primer: GACTTCGTGATCCACCTGC, the size of PCR product is about 240 ~ 260 bp) and D8SAC016868 (Forward primer: AAGTCAAAGGCCAGGGTGT; Reverse primer: TCATGTGTTTCCC AGGAATG, the size of PCR product is about 230 ~ 250 bp), located close to the SFRP1 locus. Amplification was done in 5 μl volumes with 20 ng of genomic DNA, 0.06 μmol/L of each primer (5' fluorescent-labeled primers), 0.2 mmol/L each dinucleotide triphosphate, and 0.2 U hotstar Taq polymerase under the following conditions: 94°C (hot start) for 10 min, followed by 30 cycles at 94°C for 30 sec, at 55°C for 30 sec, and at 72°C for 30 sec, with a final extension at 72°C for 10 min. PCR products were analyzed using ABI 3730 sequencer. LOH was analyzed by determining the fluorescent intensity of each allele and calculating the ratio using peak height as the following formula: LOH=Height of normal allele twoHeight of normal allele oneHeight of tumor allele twoHeight of tumor allele one
 MathType@MTEF@5@5@+=feaafiart1ev1aaatCvAUfKttLearuWrP9MDH5MBPbIqV92AaeXatLxBI9gBaebbnrfifHhDYfgasaacH8akY=wiFfYdH8Gipec8Eeeu0xXdbba9frFj0=OqFfea0dXdd9vqai=hGuQ8kuc9pgc9s8qqaq=dirpe0xb9q8qiLsFr0=vr0=vr0dc8meaabaqaciaacaGaaeqabaqabeGadaaakeaacqqGmbatcqqGpbWtcqqGibascqGH9aqpfaqaaeGabqaabaWaaSaaaeaacqqGibascqqGLbqzcqqGPbqAcqqGNbWzcqqGObaAcqqG0baDcqqGGaaicqqGVbWBcqqGMbGzcqqGGaaicqqGUbGBcqqGVbWBcqqGYbGCcqqGTbqBcqqGHbqycqqGSbaBcqqGGaaicqqGHbqycqqGSbaBcqqGSbaBcqqGLbqzcqqGSbaBcqqGLbqzcqqGGaaicqqG0baDcqqG3bWDcqqGVbWBaeaacqqGibascqqGLbqzcqqGPbqAcqqGNbWzcqqGObaAcqqG0baDcqqGGaaicqqGVbWBcqqGMbGzcqqGGaaicqqGUbGBcqqGVbWBcqqGYbGCcqqGTbqBcqqGHbqycqqGSbaBcqqGGaaicqqGHbqycqqGSbaBcqqGSbaBcqqGLbqzcqqGSbaBcqqGLbqzcqqGGaaicqqGVbWBcqqGUbGBcqqGLbqzaaaabaWaaSaaaeaacqqGibascqqGLbqzcqqGPbqAcqqGNbWzcqqGObaAcqqG0baDcqqGGaaicqqGVbWBcqqGMbGzcqqGGaaicqqG0baDcqqG1bqDcqqGTbqBcqqGVbWBcqqGYbGCcqqGGaaicqqGHbqycqqGSbaBcqqGSbaBcqqGLbqzcqqGSbaBcqqGLbqzcqqGGaaicqqG0baDcqqG3bWDcqqGVbWBaeaacqqGibascqqGLbqzcqqGPbqAcqqGNbWzcqqGObaAcqqG0baDcqqGGaaicqqGVbWBcqqGMbGzcqqGGaaicqqG0baDcqqG1bqDcqqGTbqBcqqGVbWBcqqGYbGCcqqGGaaicqqGHbqycqqGSbaBcqqGSbaBcqqGLbqzcqqGSbaBcqqGLbqzcqqGGaaicqqGVbWBcqqGUbGBcqqGLbqzaaaaaaaa@B77B@, where the longer or shorter alleles were considered to be significantly lost in HCC specimens when an LOH value ≤0.5 or ≥1.5 was observed, respectively.

### Treatment of 5-aza-2'-deoxycytidine and trichostatin A

To evaluate whether the genomic DNA methylation can contribute to the re-expression of SFRP1, both 5-aza-2'-deoxycytidine (DAC) (1000 nM) (Sigma), a demethylation reagent, and trichostatin A (TSA) (300 nM) (Wako BioProducts, Richmond, VA), an inhibitor of histone deacetylase, were employed to treat Bel7404, QGY7701 and MHCC-H cells with low expression of endogenetic SFRP1.

### Methylation-specific PCR and Bisulfite Sequencing

We treated DNA with bisulfite according to the previous description [29]. Briefly, 1 μg of genomic DNA was denatured by incubation with 0.2 M NaOH. Aliquots of 10 mM hydroquinone and 3 M sodium bisulfite (pH 5.0) were added and the solution was incubated at 50°C for 16 hrs. To analyze the DNA methylation status of the CpG islands of SFRP1 in HCCs and cell lines, Methylation-Specific PCR (MSP) was performed with genomic DNA treated by bisulfite, where specific primers for unmethylated and methylated DNA were designed within the CpG island of SFRP1, as following: Methylation (forward: AGTTAGTGTC GCGCGTTC; reversal: CCGATACCCATACCGACTC) and Unmethylation (forward: GGAGTTGGGGTGTATTTAGTTTG; reversal: CCAATACCCATACCAACTCTACA). The lengths of "M" and "U" product are 299 bp and 247 bp, respectively. In addition, DNA sequencing on PCR products was also carried out to further assess the DNA methylation status of SFRP1 promoter, where the CpG islands-enriched region within SFRP1 promoter was amplified with bisulfite-treated genomic DNA by using the primers (forward: TTTATGGGTTTGTAAGTATGATTTAGG; reversal: ACAAATTAAAC AACACCATCTTCTT). The length of product is 897 bp, and then the PCR products were inserted into pMD 18-T vector (TaKaRa Inc. Japan) for DNA sequencing on ABI 3730 sequencer.

### RNA interference using small interference RNA (siRNA)

Two siRNAs against SFRP1 were designed and chemically synthesized (Shanghai GenePharma Co., Shanghai, China) for targeting different coding regions of the gene as following: siRNA-SFRP1_888 (5'- GGCCAUCAUUGAACAUCUCtt-3' and 5'-GAGAUGUUCAAUGAU GGCCtt-3') for nt 888–909 of SFRP1; and siRNA-SFRP1_1094 (5'- GCCACCACUUCCUCAUCAUtt-3' and 5'-AUGAUGAGGAAGUGGU GGCtt-3') for nt 1094–1115 of SFRP1. In addition, a negative control, termed as siRNA_NC (5'-UUCUCCGAACGUGUCACGUtt-3' and 5'-ACGUGACACGUUCGGAGAAtt-3') was also synthesized in this study. All above siRNAs were transfected into SMMC7721 cells to observe the rescue of depressed cell growth triggered by exogenous SFRP1.

## Results

### SFRP1 was frequently down-regulated in primary HCC

**Figure 1:**
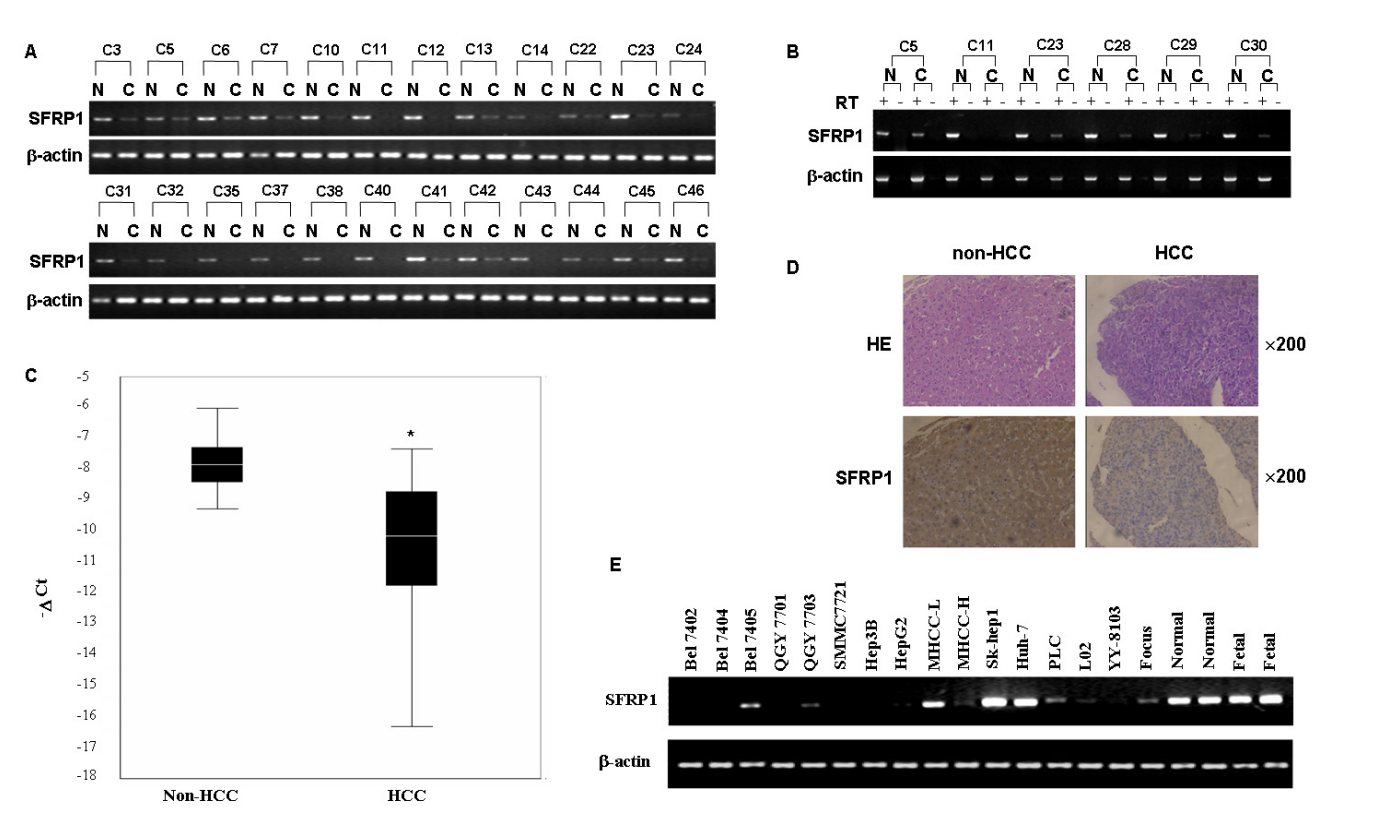
**Expression pattern of SFRP1 in HCC specimens by RT-PCR and immunohistochemical staining**. **(A) **Representative results of semi-quantitative RT-PCR of SFRP1 from 24 pairs of HCCs (C) and corresponding adjacent non-cancerous livers (N), where β-actin was employed as an internal control. Each PCR was generally performed in 32 thermal cycles and PCR products were visualized after electrophoresis through 2% agarose. The length of PCR product of SFRP1 and β-actin are 328 bp and 295 bp, respectively. **(B) **To confirm the absence of influences of genomic DNA contamination, 6 Paired HCCs and non-HCCs were selected randomly to detect the SFRP1 and beta-actin expression by RT-PCR. Experiments were performed by using RT (RT+) or no RT (RT-) in each sample. **(C) **Real time RT-PCR analysis of SFRP1 was carried out on 46 paired HCCs and adjacent non-cancerous livers. For each sample, the relative mRNA level of SFRP1 was normalized based on that of β-actin. The line within each box represents the median -ΔCt value; the upper and lower edges of each box represent the 75th and 25th percentile, respectively; the upper and lower bars indicate the highest and lowest values determined, respectively. * indicates p value <0.001. **(D) **Representative immunohistochemical staining of a pair of HCC specimen and corresponding non-cancerous liver with anti-SFRP1 antibody. The nuclei were countered stained with hematoxylin. **(E) **Expression pattern of SFRP1 was evaluated in HCC cell lines, fetal and adult normal livers through RT-PCR, where β-actin was used as a loading control.

To evaluate the transcriptional expression of SFRP1 in primary HCCs, semi-quantitative RT-PCR was employed to detect the mRNA level of SFRP1 in 120 pairs of HCC specimens and their adjacent non-cancerous liver tissues. The results showed that SFRP1 was frequently down-regulated in 48% (58/120) HCC specimens as compared with adjacent non-cancerous livers, of which the representative RT-PCRs from 24 pairs of HCC samples were showed as Figure 1A. To confirm the absence of genomic DNA contamination, 6 paired HCCs and non-HCCs were randomly selected to detect the expression of SFRP1 and β-actin by RT-PCR. The products of SFRP1 and β-actin were confirmed by DNA sequencing. The resulting data showed that no PCR products were amplified in all of samples without RT (RT-), indicating that there is no genomic DNA contamination in these RNA samples (Fig. 1B). Considering the limitation of RT-PCR method, the mRNA level of SFRP1 was also further evaluated to confirm the down-regulation of this gene in 46 informative cases (Table 1) through real time RT-PCR. Of these 46 cases, 35 (76.1%) HCCs showed at least a 2-fold reduction of the SFRP1 mRNA level as normalized by β-actin level in each sample as compared with that of the corresponding nontumorous livers. The resulting data showed that the transcriptional expression of SFRP1 was significantly reduced in HCC (p < 0.001, Fig. 1C) by comparing between tumor and non-cancerous liver groups. However, the down-regulation of SFRP1 was not statistically correlated with the gender, age (≥60 or <60), or tumor size (≥3 or <3 cm) (p > 0.05, Table 2).

**Table 2:** The expression of SFRP1 *versus *clinical features

Clinicopathological parameters		Number of patients	Down* regultaion	No Down regultaion	*X*^2^	p
Gender						
	male	40	32 (80.2%)	8 (20.0%)	2.770	0.065
	female	6	3 (50.0%)	3 (50.0%)		
Age						
	≥60	7	5 (71.4%)	2 (28.6%)	0.098	0.754
	<60	39	30 (76.9%)	9 (23.1%)		
Tumor Size (cm)						
	>3 cm	39	31 (45.5%)	8 (54.5%)	1.629	0.202
	≤3 cm	7	4 (37.5%)	3 (62.5%)		
D8S532						
	LOH+	6	5 (83.3%)	1 (16.7%)	0.361	0.548
	LOH-	40	30 (75%)	10 (25%)		
D8SAC016868						
	LOH+	3	3 (100.0%)	0	1.30	0.254
	LOH-	43	32 (74.4%)	11 (25.6%)		

*Down-regulation of SFRP1 was designed as ≤0.5 (HCC/non-HCC)

To further confirm the down-regulation of SFRP1 at protein levels, immunohistochemical staining was performed on an additional 100 pairs of HCC specimens and corresponding adjacent non-cancerous livers using tissue array (Shanghai OUTDO Biotech Co, LTD, China), where immunohistochemical-staining intensity was scored on a scale of 1+ to 3+. Interestingly, SFRP1 was found also decreased in 30 (30%) of 100 HCC specimens as compared to adjacent non-cancerous livers (p < 0.05), where the stain intensity of SFRP1 in those non-cancerous livers was generally scored to the scale of 3+. The immunohistochemical staining showed that SFRP1 was mostly anchored in cytoplasm and extracellular matrix (ECM) (Fig. 1D), in coincidence with the character of SFRP1 as a secreted protein. Furthermore, we evaluated the expression level of SFRP1 in available HCC cell lines by RT-PCR. The resulting data showed that SFRP1 was significantly expressed in Bel7405, QGY7703, MHCC-L, Sk-Hep1, HuH-7, PLC, and Focus cell lines, whereas no or weak expression of the gene was found in Bel7402, Bel7404, QGY7701, SMMC7721, Hep3B, HepG2, MHCC-H, L02 and YY-8103 cell lines (Fig. 1E). Together these findings indicated that the down-regulation of SFRP1 could be an important event in oncogenesis of HCC.

### Exogenous SFRP1 could inhibit cell growth of HCC cells

**Figure 2:**
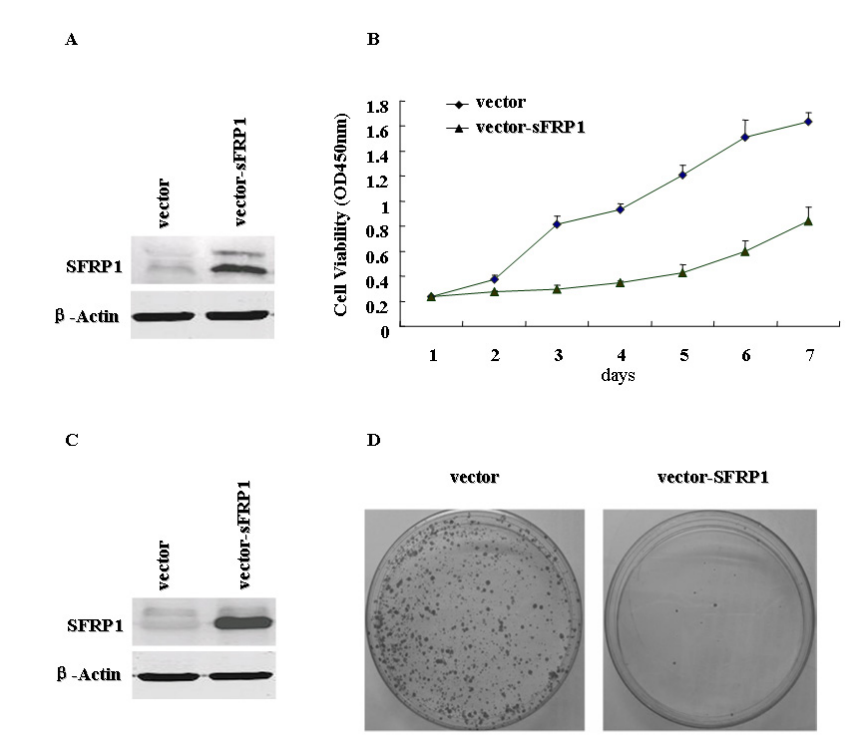
**The overexpression of SFRP1 can significantly inhibit the cell growth and colony formation of HCC cells**. **(A) **Plasmid pcDNA3.0-SFRP1 was transiently transfected into YY-8103 cells, confirmed by immunoblotting assay, where empty vector was used as control and β-actin was used as an internal reference. **(B) **Cell growth curve of YY-8103 cells with the exogenous SFRP1, which cultured in RPMI 1640 with 10% FBS. The cells transfected with the empty vector pcDNA3.0 were served as control. The experiments were repeated at least three times. The result represents the average value of triplicate wells, with standard deviation. T-test was performed to determine the statistical significance between both vector and SFRP1 experiments using SPSS software, and p<0.05. **(C) **Plasmid pcDNA3.0-SFRP1 was also transiently transfected into Hep3B cells, where the overexpression of SFRP1 was confirmed by immunoblotting assay, as compared to the control transfected by empty vector. **(D) **The colony formation of Hep3B cells was markedly inhibited as transfected with exogenous SFRP1, where the empty vector pcDNA3.0 was served as control (left). Here, after transfection for 24 h, the cells were striped and plated on 100 mm-dishes and then cultured by G418 (600 mg/ml) for 3 weeks. The dishes were stained with crystal violet solution and the number of colonies was counted from three independent experiments. The right histogram showed the colony formation efficiency, where the numbers represented the average value of three independent experiments, with standard deviation (p < 0.01, as compared with that of vector control).

**Figure 3:**
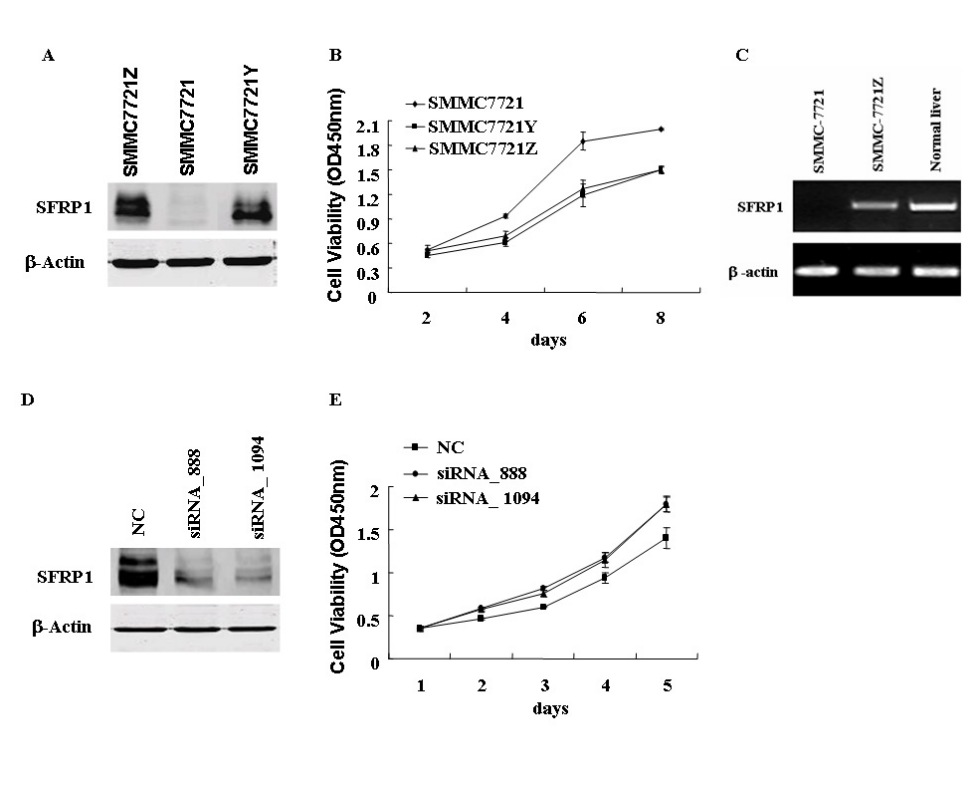
**The overexpression and RNA interference of SFRP1 can affect the cell growth of SMMC7721 cells**. (**A**) SMMC7721 cells were stably transfected by plasmid containing SFRP1, where both offspring subclones, SMMC7721Z and SMMC7721Y, were validated to be overexpressed through western blotting assay, whereas no expression of the gene was confirmed in SMMC7721-mock although transfected with the same vector. (**B**) The overexpression of SFRP1 can suppress the cell growth of SMMC7721 cells as compared with the mock. The result represents the average value of triplicate wells, with standard deviation, p < 0.01. **(C) **Analysis of SFRP1 expression level of SMMC7721Z and normal human liver through RT-PCR, where β-actin was used as a loading control. (**D**) RNA interference using both siRNA_888 and siRNA_1094 was employed to knockdown the constitutive SFRP1 in SMMC7721Z cells, where both these siRNAs were effective as compared to siRNA_NC as control, demonstrated by western blotting assay. **(E) **Cell growth of SMMC7721Z cells was promoted by both siRNA_888 and siRNA_1094, not siRNA_NC. The result represented the average value of triplicate wells, with standard deviation, p < 0.05.

To assess whether the down-regulation of SFRP1 could contribute to hepatocarcinogenesis, plasmid pcDNA3.0 with full ORF of SFRP1 under the control of the SV40 promoter were first transiently transfected into YY-8103, a HCC cell line, without the significant expression of endogenetic SFRP1 (Fig. 1E), where the empty vector pcDNA3.0 was used as control (Fig. 2A). The interesting results showed that exogenous SFRP1 can significantly inhibit the cell growth of YY-8103 cells as compared to the vector alone (Fig. 2B), suggested that SFRP1 as a Wnt pathway antagonist could play important roles in regulating negatively the cell growth of HCC-derived cells. To further test the negative effect of SFRP1 on the cell growth of HCC cells, the colony formation assay was performed on another HCC cell line, Hep3B, without the significant expression of endogenetic SFRP1, through the transient cell transfection with the same plasmids, pcDNA3.0 with full ORF of SFRP1 and empty vector (Fig. 2C). After cultured in G418 for 21 days, few colonies can be formed when the cell transfection using pcDNA3.0 with SFRP1, whereas the colony formation was still obvious as the cell transfection with empty vector alone (Fig. 2D). The dramatic reduction of colony formation from Hep3B cells further suggested that SFRP1 could be a potent inhibitor for negatively regulating the cell growth, possibly through the opposed effect of Wnt-β-catenin pathway, which was well-known as an important contributor to the oncogenesis of HCC [30-33]. To further observe the long-term effect of SFRP1 on the cell growth of HCC cells, the same plasmids were stably transfected into SMMC7721 cells, also a HCC cell line. After screening the transfected cells by western blotting assay, five stable offspring subclones were picked up and maintained. Among them, two stable subclones (SMMC7721Z and SMMC7721Y) with significant expression of exogenous SFRP1 were further evaluated by this study (Fig. 3A). The resulting data showed that SMMC7721Z and SMMC7721Y with stable expression of SFRP1 exhibited the depressed cell growth as compared to parent SMMC7721 cells without endogenous expression of the gene (p < 0.01, Fig. 3B). These transient and long-term effects of SFRP1 on different HCC cells support the notion that SFRP1 as a candidate tumor suppressor genes could be involved in hepatocarcinogenesis. To eliminate the artificial growth suppression induced by the overexpression of various genes, we compare the expression level of SFRP1 in SMMC7721Z containing exogenous SFRP1 with that of normal liver through RT-PCR (Fig. 3C). The resulting data showed that the mRNA level of exogenous SFRP1 in SMMC7721Z was not higher than that of human normal liver, implying that the growth suppression of SMMC7721Z could be induced by SFRP1 itself, not by the artificial overexpression of ectopic gene.

### Rescue of the suppressive cell growth of exogenous SFRP1 through RNA interference

To further evaluate the contribution of the down-regulation of SFRP1 to oncogenesis of HCC, two small interference RNAs (siRNAs), siRNA-SFRP1_888 for nt 888–909 and siRNA-SFRP1_1094 for 1094–1115 of SFRP1, were designed and chemically synthesized for the knockdown of SFRP1. To test the efficacy of these siRNAs, the siRNAs were transiently transfected into SMMC7721Z cells with exogenous SFRP1 because the endogenetic SFRP1 was difficult to be detected by using western blotting assay, where siRNA-NC was used as a reference control. The resulting data indicated that these two siRNAs can significantly knockdown the exogenous SFRP1, as compared with siRNA-NC (Fig. 3D). Afterward, these siRNAs were further transiently transfected into some HCC cell lines, such as MHCC-L and Sk-hep-1 cells, with the endogenetic SFRP1. However, the growth of these cells had no significant alteration, whatever promotion or suppression of cell growth, implying that those HCC cell lines could be inappropriate to evaluate the effect of SFRP1 on cell growth.

Furthermore, we still chose SMMC7721 cells to evaluate the efficiency of these siRNAs, because the above data indicated that SMMC7721 cells exhibited the response to the exogenous SFRP1. Here, these siRNAs were transiently transfected into stable offspring SMMC7721Z cells with constitutional expression of exogenous SFRP1. Interestingly, both siRNA-SFRP1_888 and siRNA-SFRP1_1094 could obviously promote cell growth of SMMC7721Z cells as compared to siRNA_NC (p < 0.05, Fig. 3E), indicated that the suppressive cell growth of exogenous SFRP1 could be rescued by the RNA interference. The data also further supported that the frequent down-regulation of SFRP1 could contribute to hepatocarcinogenesis via promoting the cell growth of given HCC cells, since the cell growth of some HCC cells was indeed negatively regulated through the expression level of SFRP1.

### LOH at the SFRP1 locus in HCCs

**Figure 4:**
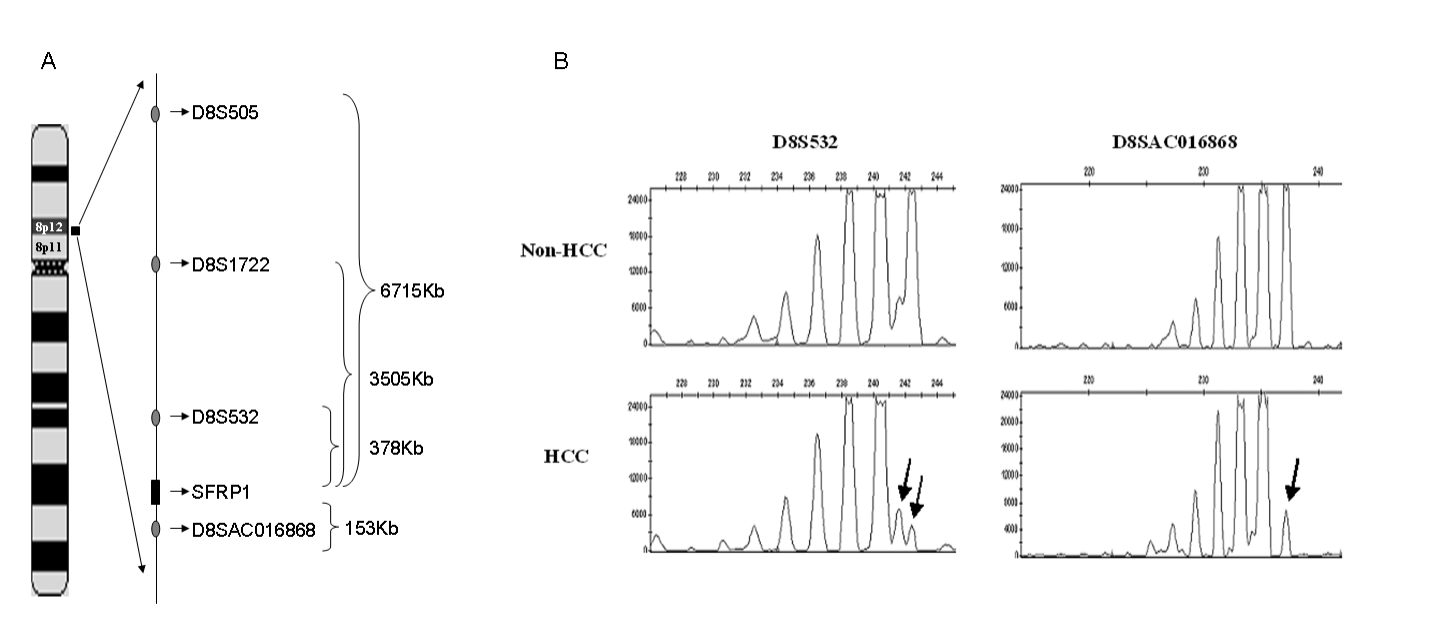
**Loss of heterozygosity (LOH) analysis on HCC samples**. **(A) **Schematic representation of the microsatellite markers of D8S532 and D8SAC016868 located on chromosome 8p11.2 flanking the SFRP1 locus. **(B)**The microsatellite markers, D8S532 and D8SAC016868, flanking the SFRP1 locus were employed to analyze the LOH on 46 pairs of HCC samples. Here, we showed a representative result of LOH from primary HCC (below) and corresponding adjacent non-cancer liver (upper), where the arrows indicated the deleted alleles in the tumor DNA.

To address whether the genetic aberrations could contribute to the down-regulation of SFRP1, genomic imbalance of the SFRP1 locus was evaluated in 46 pairs of HCC specimens with or without SFRP1 expression by using the microsatellite markers D8S532 and D8SAC016868, which were found on chromosome 8p11.2 flanking the SFRP1 locus (Fig. 4A). Among these 46 pairs of HCCs examined, LOH of these two microsatellite markers was identified in 13% (6 of 46) and 6.5% (3 of 46) HCC specimens (Fig. 4B), respectively, where a total of 8 (17.4%) HCCs was considered as involving LOH. Interestingly, SFRP1 was down regulated in seven of eight HCC specimens. However, the overall resulting data suggested that low frequency of LOH at the SFRP1 locus could not be a crucial genetic event in HCCs, although epimutation could occur by the DNA methylation on the remaining allele in those HCC samples when an allele was lost.

### DNA methylation status of SFRP1 promoter in HCC cell lines

**Figure 5:**
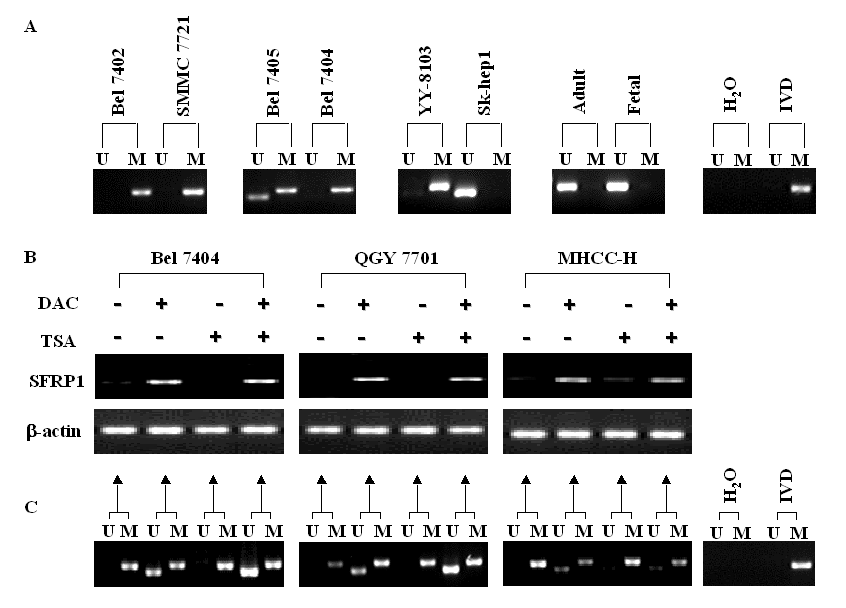
**The correlation between the expression of SFRP1 gene and DNA methylation status of SFRP1 promoter**. (**A**) DNA methylation status of SFRP1 promoter was assessed in some HCC cell lines with or without the expression of endogenetic SFRP1, as well as fetal and adult normal livers, through MSP assay with specific primers. The peripheral blood lymphocyte (PBL) DNA treated with SssI Methylase (New England Biolabs, Beverly, MA) was used as a positive control for methylation (IVD), and water was used as a negative control (H_2_O). **(B) **Bel7402, QGY7701 and MHCC-H cells without the expression of endogenous SFRP1 were treated with 5-aza-2'-deoxycytidine (DAC) and trichostatin A (TSA) alone or in combination. The expression of SFRP1 was then evaluated by RT-PCR. Untreated HCC cells were employed as control (lane 1). **(C) **The DNA methylation status of SFRP1 promoter in these cells treated by DAC and TSA were further evaluated by MSP assay and the same primers, where bisulfite-treated genomic DNA from these specimens was used as template. M, methylated; U, unmethylated.

To assess the DNA methylation level within SFRP1 promoter in these cell lines, MSP was performed on genomic DNA treated by bisulfite with the designed primers. Interestingly, the results showed that DNA hypermethylation within SFRP1 promoter indeed occurred in Bel7402, SMMC7721, Bel7404 and YY-8103 cells without the expression of endogenetic SFRP1, whereas partially or complete unmethylation of the region were found in Bel7405 and Sk-Hep1 cells, respectively (Fig. 5A). Similarly, as a control, we here evaluated the expression of SFRP1 and DNA methylation level within SFRP1 promoter in both fetal and adult livers. The resulting data demonstrated that the obvious DNA hypomethylation of the promoter was significant in the livers, in consistence with the high expression of SFRP1. The findings implied that the methylation status of SFRP1 promoter could be associated with the expression of the gene.

To further investigate whether the epigenetic events could contribute to the down-regulation of SFRP1, both the demethylation agent DAC and the histone deacetylase inhibitor TSA were employed to treat some HCC cell lines, such as Bel7404, QGY7701, MHCC-H cells, without the expression of endogenetic SFRP1 (Fig. 1E). Interestingly, the resulting data showed that SFRP1 was significantly upregulated in Bel7404, QGY7701, MHCC-H cells through the treatment by DAC, not by TSA (Fig. 5B), implying that the DNA methylation status of genomic DNA could be correlated with the dysregulation of SFRP1. To further address the relationship between the methylation status on SFRP1 promoter and the expression of the gene, MSP was performed on these three HCC cell lines to detect the methylation levels of CpG islands within SFRP1 promoter. Expectedly, MSP data suggested that the CpG islands within SFRP1 promoter could be in DNA hypermethylation in all parent cell lines, whereas the unmethylated CpG islands occurred in the cell lines as the treatment of DAC, not TSA, along with the re-expression of endogenetic SFRP1 (Fig. 5C). These findings indicated that the DNA hypermethylation of SFRP1 promoter might be responsible for the silence of SFRP1 transcription.

### DNA methylation of the SFRP1 promoter in primary HCCs

**Figure 6:**
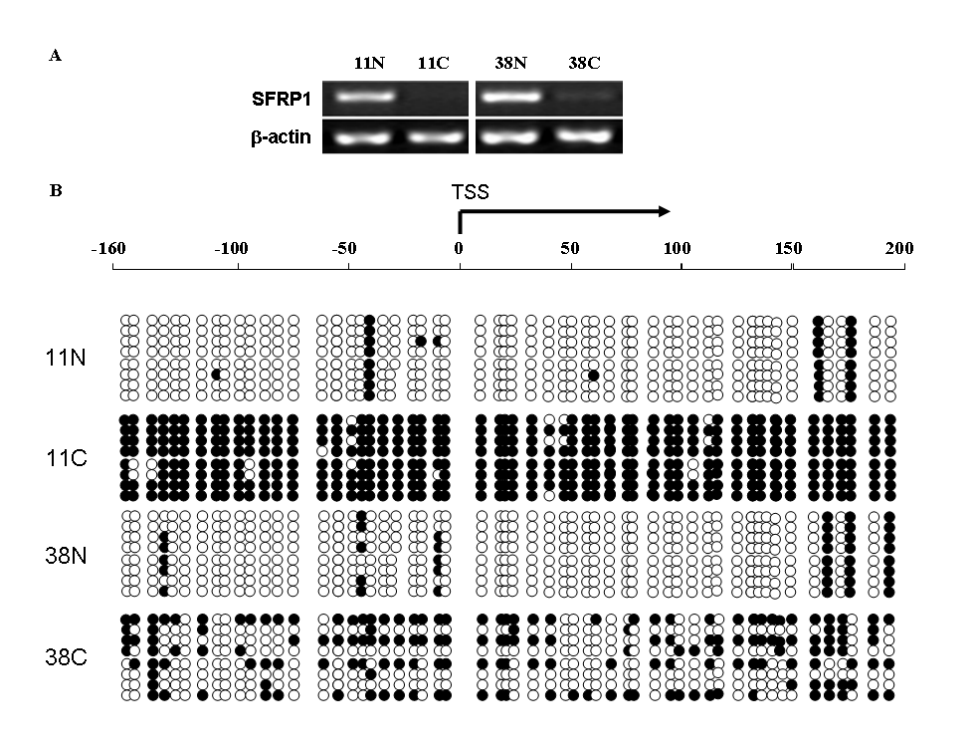
**DNA hypermethylation of SFRP1 promoter in primary HCC samples**. **(A) **Transcriptional expression of SFRP1 was analyzed by RT-PCR in two pairs of HCCs and corresponding non-cancerous livers, where β-actin was used as an internal control. **(B) **The bisulfite-treated DNA sequencing was preformed on the genomic region (-160 ~ + 200) around TSS of SFRP1, where the extent of methylation of the promoter was evaluated through DNA sequencing on 8 random clones inserted by PCR products. There were 56 CpG dinucleotides (CpGs), represented by circles, located on the region. Black and white circles represented the methylated and unmethylated CpG dinucleotides, respectively, where the methylation status was determined by bisulfite-treated DNA sequencing on the corresponding clones. C, HCCs; N, non-cancerous livers.

To quantify the DNA methylation status of SFRP1 promoter in clinical HCC samples, bisulfite-treated genomic DNA sequencing was employed to analyze the methylation level of CpG islands in three pairs of HCC specimens with the down-regulation of SFRP1 (Fig. 6A). The DNA sequencing on PCR products revealed that the methylation levels of CpG islands around the transcription start site (TSS) of SFRP1 was significantly increased in two HCC specimens, 11C and 38C, as compared to that of non-cancerous livers (p < 0.01) (Fig. 6B), whereas the methylation status in one HCC sample was not significantly changed. The resulting data suggested that the DNA hypermethylation of CpG islands around TSS of SFRP1 can partially contribute to the down-regulation of SFRP1 in some HCC specimens, although other mechanisms, such as LOH and/or other epigenetic alterations, may also contribute to the down-regulation of SFRP1 in HCCs.

## Discussion

Wnt signaling pathway is evolutionally conserved and involved in a variety of cellular processes, such as the control of cell polarity, cell fate determination, cellular proliferation, motility, morphology, axis formation, organ development, and even malignant transformation [34-37]. Interestingly, SFRP1 encodes a soluble Wnt antagonist and is located on chromosome 8p12-p11.1, which is a frequently affected region involving genetic alterations in some tumors, including breast cancer [38], colorectal carcinomas [39], and HCC [8,22]. Moreover, the down-regulation of SFRP1 has been observed in many tumors, including breast cancer [14,40], ovarian cancer [17,41], bladder cancer [13,42], mesothelioma [15], prostate cancer [18,38], colorectal cancer [12,39], and non-small cell lung cancers [26]. Recently, the frequent down-regulation of the gene was also reported in HCC [22], where 43 of 47 HCC (91.5%) exhibited the depressed SFRP1 as compared with non-cancerous livers through quantitative RT-PCR. In this study, we also found that SFRP1 was significantly downregulated in 76.1% (35/46) in HCCs by quantitative real-time RT-PCR. The lower frequency of SFRP1 down-regulation than the previous report [22] could be ascribed to the heterogeneity of hepatocellular carcinoma due to etiology, ethnics, etc. Moreover, SFRP1 was found to be significantly decreased in 30 (30%) of 100 HCC specimens on tissue array at protein levels. These findings suggested that the down-regulation of SFRP1 could be involved in some HCCs, although the down-regulation of the gene was not correlated with the etiology, gender, and tumor size. In general, our findings above implied that the frequent down-regulation of SFRP1 mapped onto chromosome 8p12-p11.1, a major LOH region in human HCC, could be an important event in oncogenesis of HCC.

In this study, we evaluated the DNA methylation status of SFRP1 promoter in many HCC cell lines and primary HCC specimens through MSP and bisulfite-treated genomic DNA sequencing. In these HCC cell lines examined, the unmethylated CpG islands within SFRP1 promoter was found in Sk-Hep1 and Bel-7405 cells with the expression of SFRP1 via MSP, whereas the significant hypermethylation of the region occurred in Bel7402, SMMC7721, Bel7404, YY-8103, QGY7701, and MHCC-H cells without SFRP1 transcription. The results supported that the DNA methylation status of SFRP1 promoter can correlate with the transcriptional expression of the gene. Actually, two of three HCC specimens indeed exhibited the significant hypermethylation of SFRP1 promoter, in consistency with the previous description [22], suggesting that the hypermethylation of SFRP1 promoter could be an important event for the down-regulation of the gene in HCC.

However, epi-mutation on a remaining allele as an allele was lost in HCC specimens could not be crucial event in hepatocarcinogenesis, because low frequency of LOH occurred at the SFRP1 locus. In this study, we used additional microsatellite markers D8S532 and D8SAC016868 that are proximal to the SFRP1 locus, not D8S505 and D8S1722 used in the previous report [22], to evaluate allelic imbalance of the SFRP1 locus. The two microsatellite markers (D8S532 and D8SAC016868) were closer to the SFRP1 locus in both flank, not one flank, than others reported by Shil YL *et al *[22] based on the genomic information (see Fig. 4A). Both D8S532 and D8SAC016868 are located in the upstream or downstream of SFRP1, only a 378 kb or 153kb distance from SFRP1 locus, respectively. Our result showed that only 13% (6 of 46 HCCs) and 6.5% (3 of 46 HCCs) exhibited the allelic imbalance, where seven of eight HCC specimens with LOH showed the depressed expression of SFRP1. As known, the ideal reference for LOH analysis is a non-pathologic tissue of the same patients, such as blood cells. In this study, it is possible that the under-estimation of LOH ratio could occur when non-tumor livers with pathological lesion were used as control.

Interestingly, it is known that SFRP1 could control vascular cell proliferation, skeletal muscle growth and retinal ganglion cell axons growth *in vitro *or *in vivo *[23-26]. However, whether the silencing of SFRP1 could contribute to hepatocarcinogenesis remains unknown. Significantly, in this study, our data indicated that the expression level of SFRP1 as a negative regulator was crucial to the cell growth of some HCC cells. The overexpression of SFRP1 can obviously inhibit the cell growth and colony formation of YY-8103, Hep3B and SMMC7721 cells, whereas RNA inference on the exogenous SFRP1 can induce the growth of the given cells (SMMC7721), indicating that the cellular behaviors of some HCC cells could be sensitive to the expression level of SFRP1, and the significant reduction of SFRP1 could contribute to oncogenesis of HCC through promoting cell growth. On the other hand, our data also implied that SFRP1 as a tumor suppressor gene and secreted protein for inhibiting Wnt-β-catenin could be employed for cancer therapy through transfecting the gene into tumor, and injecting the active protein as surroundings niche.

## Conclusion

Our data suggested that the down-regulation of SFRP1 as a candidate tumor suppressor gene, triggered by the epigenetic and/or genetic events, could contribute to the oncogenesis of HCC.

## Abbreviations

SFRP1, secreted frizzled-related protein 1; HCC, Hepatocellular carcinoma; MSP, methylation-specific polymerase chain reaction; LOH, loss of heterozygosity.

## Competing interests

The author(s) declare that they have no competing interests.

## Authors' contributions

ZGH and JH design the study. JH and YLZ performed plasmid construction, cell culture, cell transfection and RT-PCR analysis; JH and YL performed methylation assay; XMT performed western blotting analysis; DLZ carried out the PCR experiments. JH, YLZ and ZGH wrote the manuscript. PYY participated in the design of the study. ZGH conceived of the study and coordination and revised the article. All authors read and approved the final manuscript.

## Pre-publication history

The pre-publication history for this paper can be accessed here:


